# 721 The Role of Circadian Rhythms in Burn Injuries and Outcomes

**DOI:** 10.1093/jbcr/irae036.264

**Published:** 2024-04-17

**Authors:** Sheera F Lerman, Lisa C Smith, Iman F Khan, Michelle Mei, Tomer Lagziel, Carrie A Cox, Julie A Caffrey

**Affiliations:** Department of Psychiatry and Behavioral Sciences, Johns Hopkins University, Baltimore, Maryland; Department of Nursing, Johns Hopkins Bayview Medical Center, Baltimore, MD; Department of Plastic Surgery, Johns Hopkins School of Medicine, Baltimore, MD; Johns Hopkins Burn Center, Lutherville Timonium, MD; Johns Hopkins University School of Medicine, Baltimore, MD; Department of Psychiatry and Behavioral Sciences, Johns Hopkins University, Baltimore, Maryland; Department of Nursing, Johns Hopkins Bayview Medical Center, Baltimore, MD; Department of Plastic Surgery, Johns Hopkins School of Medicine, Baltimore, MD; Johns Hopkins Burn Center, Lutherville Timonium, MD; Johns Hopkins University School of Medicine, Baltimore, MD; Department of Psychiatry and Behavioral Sciences, Johns Hopkins University, Baltimore, Maryland; Department of Nursing, Johns Hopkins Bayview Medical Center, Baltimore, MD; Department of Plastic Surgery, Johns Hopkins School of Medicine, Baltimore, MD; Johns Hopkins Burn Center, Lutherville Timonium, MD; Johns Hopkins University School of Medicine, Baltimore, MD; Department of Psychiatry and Behavioral Sciences, Johns Hopkins University, Baltimore, Maryland; Department of Nursing, Johns Hopkins Bayview Medical Center, Baltimore, MD; Department of Plastic Surgery, Johns Hopkins School of Medicine, Baltimore, MD; Johns Hopkins Burn Center, Lutherville Timonium, MD; Johns Hopkins University School of Medicine, Baltimore, MD; Department of Psychiatry and Behavioral Sciences, Johns Hopkins University, Baltimore, Maryland; Department of Nursing, Johns Hopkins Bayview Medical Center, Baltimore, MD; Department of Plastic Surgery, Johns Hopkins School of Medicine, Baltimore, MD; Johns Hopkins Burn Center, Lutherville Timonium, MD; Johns Hopkins University School of Medicine, Baltimore, MD; Department of Psychiatry and Behavioral Sciences, Johns Hopkins University, Baltimore, Maryland; Department of Nursing, Johns Hopkins Bayview Medical Center, Baltimore, MD; Department of Plastic Surgery, Johns Hopkins School of Medicine, Baltimore, MD; Johns Hopkins Burn Center, Lutherville Timonium, MD; Johns Hopkins University School of Medicine, Baltimore, MD; Department of Psychiatry and Behavioral Sciences, Johns Hopkins University, Baltimore, Maryland; Department of Nursing, Johns Hopkins Bayview Medical Center, Baltimore, MD; Department of Plastic Surgery, Johns Hopkins School of Medicine, Baltimore, MD; Johns Hopkins Burn Center, Lutherville Timonium, MD; Johns Hopkins University School of Medicine, Baltimore, MD

## Abstract

**Introduction:**

Circadian rhythms are patterns responsible for physiological and behavioral changes in living organisms that follow a 24-hour cycle. Burn injuries trigger an inflammatory response which is also regulated by the circadian system. Literature on the impact of circadian rhythms on burn injury outcomes remains scarce. However, there is evidence that the migration of cells involved in the wound healing process is circadian-regulated and that the timing of the burn injury in a 24-hour period is associated with wound healing rates in burn patients. The goal of this study was to explore the relationship between time of burn injury and burn related outcomes.

**Methods:**

A retrospective chart review was conducted of 442 adult burn patients admitted to the Burn Center between January 2015 and February 2022. Mean age on admission was 50.13 years, 34% were female and patients identified as Caucasian (57%), African American (35%) or other (8%). Mean TBSA was 12.85%. Data on clinical and demographic factors and times of injury were extracted from patients’ electronic medical records. Times of burn injury were categorized as daytime versus nighttime based on local sunrise and sunset times.

**Results:**

Fifty-seven percent of patients were burned during daytime. When comparing those who were burned at daytime to those burned at nighttime, daytime burns had lower %TBSA, shorter length of stay and lower morphine equivalent dose during their admission to the burn center. In a regression analysis predicting morphine equivalent dose, time of burn remained a statistically significant predictor, even after controlling for %TBSA, length of stay, gender, race, and age.

**Conclusions:**

Our study underscores the pivotal role of burn injury timing, revealing that nighttime burns are correlated with adverse outcomes, even after accounting for other contributing factors. This insight has profound implications for optimizing burn care and developing targeted intervention strategies. While it is possible that nighttime burns are more severe and thus are associated with poorer outcomes, we found that even when controlling for burn size, nighttime burns have higher inpatient opioid requirements. This is consistent with evidence from human and animal models suggesting that the timing of a burn injury has a unique impact on circadian rhythm, which regulates a host of immune-related responses and can influence the healing process and opioid metabolism.

**Applicability of Research to Practice:**

Recognizing the time of burn injury as a significant risk can enhance clinical assessment protocols, aiding in the identification of high-risk patients. It is crucial to pursue further research to understand the biological mechanism through which timing of injury interacts with circadian function to influence burn recovery.

